# American Medical Association—Proceedings of General Session

**Published:** 1880-06

**Authors:** 


### AMERICAN MEDICAL ASSOCIATION—PROCEEDINGS OF GENERAL SESSION.

FIRST DAY—JUNE 1.—The thirty-first annual session of the American Medical Association convened in the Young Men's Christian Association Hall, in New York city, at 11 A. M., and was called to order by the President, Dr. Lewis A. Sayre, of New York City. The Secretary, Dr. Wm. B. Atkinson, of Philadelphia, was in his seat. After prayer by Rev. Dr. Wm. F. Morgan, of St. Thomas' Church, Dr. T. Gaillard Thomas, chairman of the Committee of Arrangements, delivered a most appropriate address of welcome.

Protest against registering delegates from the United States Navy and certain delegates from Alleghany county, N. Y., were referred to the Judicial Council.

On motion, by Dr. Wm. Brodie, of Michigan, a vote of thanks was tendered to the President for his able address, a copy requested for publication, and the recommendations which it contained were referred to a committee of five to report upon during the present meeting.

Dr. Sayre, who has been sick for some time, was compelled to retire; and Dr. Beverly Cole, of San Francisco, California, one of the Vice Presidents, took the chair.

A number of distinguished medical gentlemen from different States were then elected members by invitation, and were accordingly announced.

Dr. Montrose A. Pallen, from the Committee on Arrangements, announced that provision had been made for a temporary Section on Disease of Children.

Dr. Samuel D. Gross, of Philadelphia, after some touching remarks, moved that the Association tender to Dr. Sayre and his family their warmest sympathy in their sad bereavement by the death of Dr. Chas. H. H. Sayre, which motion was carried by a rising vote.

Dr. E. Seguin, of New York city, Secretary of the Foreign Delegation, etc., presented the report of the delegation on the progress of medical international uniformity as to weights and measures. On motion of Dr. Wm. Brodie, of Detroit, Mich., the report was ordered to be printed and to be laid on the desks of the members to-morrow morning.

Dr. Austin Flint, of New York, chairman of the Committee on Prize Essays, reported that the committee did not feel warranted in awarding a prize to any of the several essays presented..

After some routine announcements, the Association adjourned until 10 A.M. to-morrow.

In the evening, the entertainments consisted of a promenade concert and reception at the Academy of Music.

SECOND DAY—JUNE 2.—The meeting was called to order by the President.

On motion of Dr. James T. White, of Buffalo, N. Y., the appropriation of $200, as asked for by Dr. N. S. Davis, of

Chicago, Ill., to be expended in instruments to investigate the subject of ozone, was voted.

Dr. J. S. Lynch, of Baltimore, Md., chairman of the Section on Practice of Medicine, delivered his report.

The report was referred to the Committee on Publication.

Dr. W. T. Briggs, of Nashville, Tenn., then delivered his address as chairman of the Section on Surgery and Anatomy.

Referred to the Committee on Publication.

The following resolutions relating to prize essays were adopted:

(1) Four annual prizes of $250 each shall be awarded at the close of the second year after announcement for strictly original compositions.

(2) Chairman of the Sections on Practice of Medicine, etc., Surgery and Anatomy, Obstetrics, etc., and State Medicine, must annually appoint a competent committee of three members who shall select and publicly announce for competitive investigation and report a subject belonging to one or other of the branches of medicine included in the title of the section.

(3) These chairmen shall also annually appoint a Committee of Award, consisting of three experts, and if any one of the essays presented to this Committee of Award shall be found worthy, the committee is to recommend the same to the Association.

(4) All essays shall be in the hands of the chairman of the respective Committees of Award by January 1st, preceding the meeting of the Association at which reports are to be made.

(5) All prize essays are the property of the Association.

(6) The names of the authors of the competing essays shall be kept secret from the committees by such means as the latter may provide.

(7) Membership in either of the two committees shall not debar from membership in the other; nor shall membership in the Committee of Selection exclude a member from the privilege of offering a competitive essay.

Adjourned.

Third Day—June 3.—The report of the Committee on Nominations was presented by the chairman, Dr. W. G. Baldwin, of Montgomery, Ala., as follows:

*President*—Dr. John T. Hodgen, of St. Louis, Mo.

*Vice-Presidents*—Drs. W. H. Anderson, of Mobile, Ala.; Levi G. Hill, of New Hampshire; Henry T. Holton, of Vermont; H. Carpenter, of Oregon.

*Permanent Secretary*—Dr. W. B. Atkinson, of Philadelphia, Pa.

*Treasurer*—Dr. R. Dunglison, of Philadelphia, Pa.

*Librarian*—Dr. William Lee, Washington, D. C.

*Chairman of the Section on the Practice of Medicine, Materia Medica and Physiology*—Dr. Charles Denison, of Colorado. *Secretary*—Dr. T. A. Ashby, of Maryland.

*Chairman of the Section on Surgery and Anatomy*—Dr. H. McGuire, of Richmond, Va. *Secretary*—Dr. D. A. Eve, of Tennessee.

*Chairman of the Section on Obstetrics and Diseases of Women*—Dr. James R. Chadwick, of Boston, Mass. *Secretary*—Dr. J. Taber Johnson, of Washington, D. C.

*Chairman of the Section on Medical Jurisprudence and State Medicine*—Dr. J. T. Reeve, of Wisconsin. *Secretary*—Dr. R. G. Young, of Arkansas.

*Chairman of the Section on Opthalmology, Otology and Laryngology*—Dr. D. S. Reynolds of Kentucky. *Secretary*—Dr. S. M. Burnett, of Washington, D. C.

*Members of the Judicial Council, to fill vacancies*—Dr. J. M. Bartlett, of Wisconsin; F. Staples, of Minnesota; D. R. Wallace, of Texas; J. S. Billings, of U. S. Army; J. H. Warren, Massachusetts, and A. Woodward, Vermont.

The next meeting of the Association is to be held in the city of Richmond, Va., on the *first Tuesday* in May, 1881.

*Chairman of Committee of Arrangements*—Dr. F. D. Cunningham, of Richmond, Va.

The Committee further recommended that the Committee on Necrology and the membership of the Section on Medical Jurisprudence, State Medicine and Public Hygiene remain as now constituted. The report was adopted unanimously.

Dr. Denison offered his resignation, which was accepted, and referred to the Committee on Nominations.

Dr. Bronson, of Massachusetts, offered the following preamble and resolution, which was adopted:

*Whereas*, The published proceedings of various sections of the Association does not receive the practical expression desired, and does not represent the labors of its members; and whereas, the members of the Association have long felt that the present mode of introducing the Transactions to the profession has been unsatisfactory to all concerned in the advancement of medical science; theretore.

*Be it Resolved*, That a committee of five be appointed by the chair to report at the next session regarding the practicability of formulating all the proceedings in journalistic form, as recommend by the President in his Annual Address. Carried.

The Report on Sanitaria, presented by Dr. H. I. Bowditch, of Massachusetts, was read by title, and referred to the Section on Public Hygiene.

Dr. J. S. Billings, of Washington, D. C., reported on *The Catalogue of the National Library*, that Congress had made provisions for the publication of the volumes, of which the first would probably be published in July, 1880, and the second in July, 1881.

The following amendments to the Constitution, proposed by Dr. John H. Rauch, were adopted.

Article II, second paragraph, after "Army and Navy," insert "and the Marine Hospital Service of the United States."

Article II, fourth paragraph, at the end insert "the Marine Hospital Service of the United States shall be entitled to one delegate."

*Section on Diseases of Children*—Dr. S. C. Busey, of Washington, offered an amendment to the By-Laws, making provision for a new section to be designated as Section VI, Diseases of Children. Adopted, and referred to the Committee on Nominations.

The report of the Metric Executive Committee being next in order, it was read by Dr. Atkinson, who moved its adoption. Discussion followed, which was participated in by Drs. Brodie and Fairbanks, of Michigan, and Bronson of Massachusetts, who opposed it, and Cole, of California, Hunt, of New Jersey, Antisell, of D. C., Lyons, of Connecticut, in favor of the report, which was adopted by the Association.

The following were the propositions recommended by the Committee and adopted by the Association:

*First.* It recommends the teaching and practice of the metric system in medical colleges, clinics, dispensaries, etc.

*Second.* It charges its Executive Metric Committee with the duty to report annually on the above institutions which teach, and those which do not teach the metric system.

*Third.* It authorizes said committee to enter into communication with the Metric Committee of the British Medical Association, in order to concert such plans as may render the use of the metric system simultaneous and uniform in both countries.

Dr. Theophilus Parvin, Chairman; Dr. Edward Seguin Secretary; Drs Edward Wigglesworth and F. R. Weist, Executive Metric Committee.

Dr. W. M. Beach, of Ohio, offered a resolution making provision for the appointment of a committee of five, whose duty it should be to endeavor to secure for the medical staff of the army and navy a social position co-equal with that for like grades in any department of the service. Adopted.

*Report from the Judicial Council—Whereas,* A protest without signature, against the registration of the delegates from the Medical Staff of the U. S. Navy, and unaccompanied by charges, had been placed in the hands of the Judicial Council; therefore,

*Be it Resolved,* That the protest against the registration of delegates from the Medical Department of the U. S. Navy is not sustained, and therefore there was no cause for action thereon. Adopted.

*Resolved,* That the Hannibal Medical Society of Missouri is not entitled to representation in the American Medical Association, because it is not in affiliation with its own State Medical Society. The protest, therefore, was sustained.

The President announced the following committees: To carry out Dr. Bronson's resolutions offered in the early part of the session: Drs. W. W. Dawson, Ohio; J. R. Bronson, Mass.; W. H. Pancoast, Pa.; N. C. Husted, N. Y.; J. S. Green, N. J. On the President's Address, Drs. Chaille, La; S. D. Gross, Pa.; J. S. Weatherly, Ala.; J. R. Bronson, Mass; and W. R. Gillette, N. Y. To report in 1881.

Dr. J. M. Toner, of Washington, D. C., then read the Report of the Committee on Publication.

Delegates to the Canada Medical Association, Drs. C. N. Brush, Buffalo, N. Y.; James R. Leaming, New York, N. Y.; D. H. Goodwillie, New York, N. Y.; Wm. Brodie, Detroit, Mich.; W. B. Ulrich, Pittsburg, Pa.

Delegates to European Medical Societies, Drs. R. Beverly Cole, Cal.; Benj. Lee, Pa., M. A. Pallen, N. Y; and L. D. Bulkley, N. Y.

The report of the Librarian stated that the library contained 1,302 distinct titles, constituting 3,258 volumes. The Librarian asked for $200 with which to carry on the work of the library. The report was accepted, ordered entered upon the minutes, and an appropriation for the amount of money named granted.

Adjourned.

Fourth Day—June 4.—The following were appointed as Committee on Dr. Beach's resolutions concerning the social position of the members of the medical staff of the United States Navy: Drs. Wm. M. Beach, London, Ohio; C. Goodbroke, Clinton, Ill.; H. McGuire, Richmond, Va.; William S. Briggs, Nashville, Tenn.; D. W. Yandell, Louisville, Ky.

A communication from Dr. Frederick Horner, Jr., U. S. Navy, containing a resolution adopted by the Medical Society of Virginia, instructing its delegates to the American Medical Association to ask that body to take the necessary steps for establishing a mutual medical aid association, was received favorably and referred.

A communication from the Philadelphia County Medical Society, concerning the abuse of medical charities, was received and referred to a special committee, appointed by the chair, consisting of Drs. B. Lee, H. G. Piffard, S. W. Gross and J. W. Green.

A resolution was offered by Dr. A. L. Carroll, of the Richmond County Medical Society of New York. *Resolved*, That societies which admit irregular practitioners to membership be debarred from representation in the American Medical Association. Referred to the Judicial Council.

The Treasurer's report showed receipt of $5,024 ; balance in the treasury $579.59. The report was received and referred to the Committee on Publication.

Dr. Pratt, of Michigan, offered a resolution instructing the Committee of Arrangements to publish one programme of the work to be done in general session at the next annual meeting during the whole four days, instead of a daily programme, which was adopted.

Dr. Pratt also offered the following resolution, which was adopted, That the Committee of Arrangements be instructed to so plaçe the proposed amendment to the Code of Medical Ethics upon the programme that it shall be made the special order at 10:30 A M. of the second day's general session, and that it shall remain as a special order until disposed of.

The Committee on Nominations completed its report by offering the following :

*Assistant Secretary*—Dr. J. G. Cabell, of Virginia.

*Committee of Arrangements*—Drs. Cunningham, McGuire, and Cullen, of Richmond, Va., with power to select others to make a committee of seven.

*Committee of Publication*—The same as last year. Drs. W. B. Atkinson, T. M. Drysdale, William Lee, R. G. Dunglison, Albert Fricke, S. D. Gross, and Caspar Wistar, all of Philadelphia.

*Chairman of the Section on Practice of Medicine*—Dr. William Pepper, of Philadelphia.

*Chairman of the Section on Diseases of Children*—Dr. A. Jacobi, of New York; *Secretary*—Dr. W. H. Bradford, of Boston.

The committee also recommend that the following resolutions, passed by the Association last year at Atlanta, be recognized as remaining in force until further orders by the Association:

"That the Committee of Publication be instructed to advertise for proposals to publish the Transactions of this Association in six of the largest cities of the Union, and that the contract be awarded to the lowest and best responsible bidder.

"That the Treasurer be instructed to deposit the funds of the American Medical Asiociation, monthly, as Treasurer, in the name of the Association, in the Farmers' and Mechanics' Bank, of Philadelphia."

The report was read and adopted.

Dr. H. G. Piffard, of New York, offered the following resolution, which was referred to the Judicial Council: That the Richmond County Medical Society of New York, which has members who have been irregular practitioners of medicine, be debarred from representation in the American Medical Association.

Dr. Pallen moved a reconsideration of the vote by which the resolution relating to the printing of the Transactions, reported by the Committee on Transactions, was adopted. Carried.

Dr. Pallen then moved that the publication of the Transactions for this year be assigned to the Collins Printing House, as last year, without advertising. Carried.

On motion by Dr. Marcy, of Massachusetts, an honorarium of $1,000 was granted the permanent Secretary.

Dr. Hibbard, chairman of the Section on Medical Jurisprudence and State Medicine, offered the following resolutions as sent up from the Section:

(1) Endorsing the National Board of Health.

(2) That the Association recommend medical schools to establish a Chair of State Medicine as part of the regular curriculum.

(3) That the name of the Section hereafter shall be "Section on State Medicine."

(4) That the Committee on Prize Essays shall be Drs. S. E. Chaille, of Louisiana; J. L. Cabell, of Virginia; and A. N. Bell, of New York.

The resolutions were all adopted.

The Association was then declared adjourned, to meet in the city of Richmond, Va., on the first Tuesday in May, 1881.—*Virginia Medical Monthly.*

